# Case report: Unusual viral evolutions following antiviral therapies in a patient with concurrent hepatitis B virus and hepatitis C virus infection

**DOI:** 10.3389/fmed.2023.1136111

**Published:** 2023-02-15

**Authors:** Yi-Tse Su, Ming-Ling Chang, Yun-Fan Liaw

**Affiliations:** ^1^Liver Research Unit, Chang Gung Memorial Hospital, Taoyuan, Taiwan; ^2^Department of Medicine, College of Medicine, Chang Gung University, Taoyuan, Taiwan

**Keywords:** chronic hepatitis B, chronic hepatitis C, co-infection, reactivation, entecavir

## Abstract

Concurrent hepatitis B virus (HBV) and hepatitis C virus (HCV) infection is not uncommon as the two viruses shared the similar transmission routes. HCV is usually the dominant virus to suppress HBV, and HBV reactivation may occur during or after the course of anti-HCV treatment. By contrast, HCV reactivation after anti-HBV therapy in the concurrent HBV- and HCV-infected patients was rarely noted. Here, we reported the unusual viral evolutions of a patient with concurrent HBV and HCV infection, in whom HCV reactivation occurred during the entecavir therapy to rescue the severe HBV flare, while the following anti-HCV combination therapy with pegylated interferon and ribavirin elicited the second HBV flare despite sustained virological response to HCV infection, and further entecavir therapy healed the flare.

## Introduction

Concurrent infection with hepatitis B virus (HBV) and hepatitis C virus (HCV) infection is not uncommon in highly endemic areas as both HBV and HCV infections share the common transmission routes (1). The patients dually infected with HCV and HBV carry a higher risk of developing liver cirrhosis and hepatocellular carcinoma than those with either infection alone (2). The primary goal of the treatment of concurrent HCV and HBV infection is to eliminate or permanently suppress both viruses, and the dominant virus should be identified and treated first (3). HCV is usually the dominant virus (4) as it inhibits HBV gene expression and replication by its core protein (5), but reciprocal suppression is also possible (4). HBV reactivation following direct-acting antiviral (DAA) anti-HCV therapy thus has been reported in many patients with concurrent HBV and HCV infection (1). By contrast, HCV reactivation following anti-HBV therapy is never reported in the patients concurrently infected with HBV and HCV, although we ever noticed an episode of HCV reactivation in a dually-infected patient who achieved HBV viral response following lymphoblastoid interferon therapy (6).

Here, we reported an unusual case with concurrent HBV and HCV infection, in whom HCV reactivation occurred during entecavir therapy for the severe HBV flare, while the subsequent pegylated interferon (pegIFN)-based therapy for HCV reactivation elicited the second HBV flare despite sustained virological response (SVR) to HCV infection, and the HBV flare was successfully controlled by another entecavir therapeutic course.

## Case presentation

**FIGURE 1:**
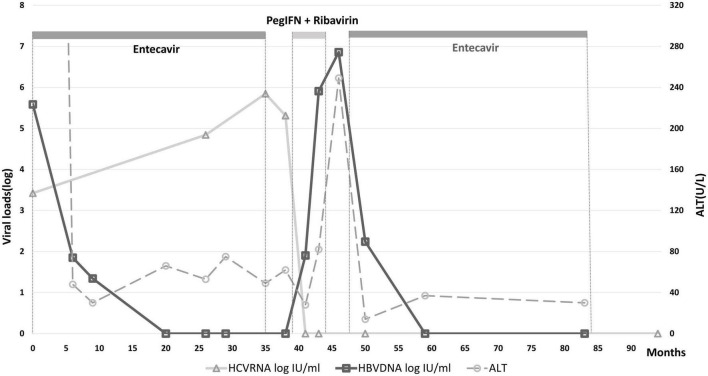
Longitudinal changes of HBV DNA, HCV RNA, and ALT levels of the HBV and HCV-coinfected patient. PegINT, pegylated interferon.

A 37-year-old male treatment-naive patient with chronic HBV (genotype B) and HCV (genotype 3) infection was treated with entecavir for severe HBV flare, presented with alanine aminotransferase (ALT) of 2,114 U/L, total bilirubin of 10.1 mg/dL, HBV DNA of 3.84 × 10^5^ IU/ml and HCV RNA of 2.65 × 10^3^ IU/ml, as shown in Table 1. He denied any prior alcohol consumption or drug abuse. Sonography disclosed a non-cirrhotic liver. The clinical course is shown in Figure 1, ALT and bilirubin levels progressively declined after entecavir therapy. The 3-year therapy was terminated in March 2016, when bilirubin level was normal and HBV DNA level was undetectable for over 1 year. However, the ALT levels were rarely under the upper limit of normal (ULN) (36 U/L) during the therapeutic course and was 49 U/L at the end of therapy (EOT), when HCV RNA level (7.02 × 10^5^ IU/ml) was > 2 log higher than its baseline level. Two months later, a combination therapy with 24-week PegIFN α-2a and ribavirin was initiated since May, 2016 and achieved rapid, early and sustained HCV virological responses. ALT levels decreased progressively in the first 12 weeks to a nadir (ALT: 28 U/L) but increased progressively in the last 12 weeks during the therapy, with HBV DNA and ALT levels of 8.08 × 10^5^ IU/ml and 82 U/L, respectively, at EOT, and increased to 7.23 × 10^6^ IU/ml and 249 U/L, respectively, at 3 months after EOT of anti-HCV therapy. He received second course of 3-year entecavir therapy to treat the second HBV flare. HBV DNA levels decreased progressively and became undetectable within 1 year and HCV RNA levels were never detectable again. The ALT levels were mostly under ULN during the entecavir therapy.

**TABLE 1:** Baseline characteristics of the patient.

Biochemistry	Hb (g/dl)	PLT (1000/uL)	P.T INR	DB (mg/dL)	TB (mg/dL)	ALK-P (U/L)	γ -GT (U/L)	AST (U/L)	ALT (U/L)	Alb (g/dL)	AFP (ng/ml)
	13.9	200	1.4	5.5	10.1	149	213	1,622	2,114	4.23	16
**Viral markers**	**HBsAg**	**Anti-HBs Ab**	**HBeAg**	**Anti-HBe Ab**	**HBV DNA**	**IgM anti-HBc Ab**	**HBV genotype**	**Anti-HCV Ab**	**HCV RNA**	**HCV genotype**	**IgM anti-HAV Ab**
	P	N	N	P	3.84 × 10^5^ IU/ml	N	B	P	2.65 × 10^3^ IU/ml	3	N

Hb, hemoglobin; PLT, platelets; P.T, prothrombin time; INR, international normalized ratio; DB, direct bilirubin; TB, total bilirubin; ALK-P, alkaline phosphatase; γ-GT, gamma glutamyltransferase; AST, aspartate aminotransferase; ALT, alanine?aminotransferase; Alb, albumin; AFP, alpha-fetal protein; HBsAg, hepatitis B surface antigen; Anti-HBsAb, anti-hepatitis B surface antibody; HBeAg, hepatitis B e antigen; Anti-HBe Ab, anti-hepatitis B e antibody; IgM-anti-HBcAb, immunoglobulin M anti-hepatitis B core antibody; anti-HCV Ab, anti-hepatitis C virus antibody; IgM anti-HAV Ab, immunoglobulin M anti-hepatitis A virus antibody; P, positive; N, negative.

## Discussion

The clinical course of the current case demonstrates HCV reactivation during anti-HBV therapy followed by HBV reactivation during anti-HCV therapy. These reciprocal changes reflect the reciprocal suppressive effects between HBV and HCV (4). The case is unique in that initially he suffered from the HBV-dominant severe hepatitis, which was rescued by entecavir. Anti-HCV therapy was not prescribed simultaneously because DAA therapy was not available until 2017 in Taiwan and peg-IFN-based therapy is contraindicated for severe hepatitis (3). Interestingly, the suppressive effect of HBV on HCV replication, although less effective (4), was evidenced by the increase of HCV level during the first entecavir therapeutic course. In contrast to HBV, HCV infection rarely induces severe acute exacerbation (7), that HBV is the most likely etiology of severe hepatitis flare in this patient. However, in the dually infected patient with efficient HBV suppression, the possibility of HCV-related hepatitis cannot be excluded even the ALT levels were only mildly elevated. During 24-week pegIFN-based therapy to treat HCV infection, the mildly elevated ALT levels returned to normal in the mid-term of therapy but elevated progressively afterward coinciding with the emergence of HBV flare. Indeed, although the pegIFN-based therapy might be effective for treating chronic concurrent HBV and HCV infection (2), HBV reactivation did occur while the patient achieved an SVR to HCV infection. It reflected the suppressed effect of HCV on HBV replication. In the era of DAA, which has no effect like Peg-IFN to suppress HBV replication, the risk of HBV reaction in concurrent HBV and HCV-infected patients with cured HCV infection is even higher and warrants further caution (8). We want to stress that entecavir withdrawal flare can occur after ceasing entecavir for few months (9), and pegylated interferon can only suppress HBV a few log IU/mL (10) and elicit immune modulation (9) may all account for the second HBV flare. Further anti-HBV therapy such as restarting entecavir should be prescribed as soon as indicated to prevent foreseeable complications including hepatic decompensation and even mortality (11). In conclusion, dually HBV and HCV-infected case treated with severe HBV flare may develop HCV reactivation which demands special caution, given that anti-HCV therapy potentially elicit further HBV flare.

## Data availability statement

The data analyzed in this study is subject to the following licenses/restrictions: The data that support the findings of this study are available on request from the corresponding author. Requests to access these datasets should be directed to M-LC, mlchang8210@gmail.com.

## Ethics statement

The studies involving human participants were reviewed and approved by Chang Gung Memorial Hospital. Written informed consent to participate in this study was provided by the participants’ legal guardian/next of kin. Written informed consent was obtained from the patient for the publication of any potentially identifiable images or data included in this article.

## Author contributions

Y-TS drafted the manuscript and analyzed the data. M-LC and Y-FL drafted the manuscript and critically revised it for intellectual content. All authors read and approved the final version of the manuscript.
